# Supplementary material for: A Comprehensive Assessment of Ultraviolet-Radiation-Induced Mutations in Flammulina filiformis Using Whole-Genome Resequencing
Source: J Fungi (Basel). 2024 Mar 20;10(3):228. doi: 10.3390/jof10030228 (PMC10971301; doi:10.3390/jof10030228)
Supplement: Supplementary file 1 [file jof-10-00228-s001.zip › Supplementary Material S8/KEGG annotation/out/64381550635650.os/KO/out_map/map04141.html]

KEGG PATHWAY: Protein processing in endoplasmic reticulum - Reference pathway


|  |  |
| --- | --- |
| **Protein processing in endoplasmic reticulum - Reference pathway** |  |

[
Pathway menu
| Organism menu
| Pathway entry
| Show description
| User data mapping
]

The endoplasmic reticulum (ER) is a subcellular organelle where proteins are folded with the help of lumenal chaperones. Newly synthesized peptides enter the ER via the sec61 pore and are glycosylated. Correctly folded proteins are packaged into transport vesicles that shuttle them to the Golgi complex. Misfolded proteins are retained within the ER lumen in complex with molecular chaperones. Proteins that are terminally misfolded bind to BiP and are directed toward degradation through the proteasome in a process called ER-associated degradation (ERAD). Accumulation of misfolded proteins in the ER causes ER stress and activates a signaling pathway called the unfolded protein response (UPR). In certain severe situations, however, the protective mechanisms activated by the UPR are not sufficient to restore normal ER function and cells die by apoptosis.

|  |  |
| --- | --- |
| Reference pathway | 100% |
